# Artemether treatment improves islet function and metabolic homeostasis in diabetic nonhuman primates

**DOI:** 10.1111/1753-0407.13347

**Published:** 2023-01-09

**Authors:** Wei Fu, Jiang Hongwei, Jin Li

**Affiliations:** ^1^ State Key Laboratory of Genetic Engineering, School of Life Sciences and Institute of metabolism and integrative biology Fudan University Shanghai People's Republic of China; ^2^ Department of Endocrinology The First Affiliated Hospital and Clinical Medicine College, Henan University of Science and Technology Luoyang People's Republic of China; ^3^ National Center for Clinical Research of Metabolic Diseases Luoyang Center for Endocrinology and Metabolism Luoyang People's Republic of China; ^4^ Diabetic Nephropathy Academician Workstation of Henan Province Luoyang People's Republic of China

**Keywords:** artemisinins, diabetes, nonhuman primates

## Abstract

**Highlights**

- Artemether increased the serum concentration of c‐peptide and decreased the usage of insulin in the diabetic monkey.
- Artemether downregulated the serum concentration of total cholesterol and low‐density lipoprotein cholesterol in the diabetic monkey.
- Artemether did not induce dramatic changes of the concentration of liver or kidney damage markers in the serum.

## 1 INTRODUCTION

The insulin insufficiency induced by loss or dysfunction of pancreatic β cells is one of the major causes of diabetes. To rescue the insulin insufficiency, many efforts have been made to develop specific therapies to improve the function of β cells via inducing regeneration. The proposed strategies include induction of β cell proliferation^1^, ^2^ or transdifferentiation of other cell types into insulin‐producing cells.^3^, ^4^

The strategy of transdifferentiation has been validated as a realistic method to rescue insulin insufficiency.^5^, ^6^, ^7^, ^8^ In addition, we have reported that the antimalaria artemisinin drugs induced transdifferentiation of pancreatic α cells into insulin‐producing cells.^9^ The therapeutic effects of artemisinins on the insulin insufficiency and metabolic disorders of rodent diabetic models, as well as the underlying mechanism, have been broadly validated in multiple independent studies.^10^, ^11^, ^12^, ^13^, ^14^, ^15^, ^16^, ^17^, ^18^

In contrast, van der Meulen et al and Ackermann et al challenged the findings about artemisinins with several models that were not employed by the original studies. Based on the negative results derived from these studies, the authors claimed that artesunate treatment may exert no or even detrimental effects on the healthy islets of mice in vivo or ex vivo.^19^, ^20^ Despite many issues in the aspect of methodology, we still appreciated the attempt to corroborate our findings with different assays.

As far as we understood, the most outstanding issue the community is interested in is whether artemisinins can be used as drugs for diabetic patients. To this end, it is pointless to debate the choice of vehicles, the methods to dissolve artemisinins, or the way to quantify transdifferentiation in rodent animal models. Also, it will only further confuse the community if we presented another piece of data showing artemether induced α‐to‐β transdifferentiation in a specific rodent model. In addition, the well‐known differences between primates and rodent animals in islet biology undermined the importance of further testing artemisinins on other rodent diabetic models. Therefore, we were evaluating the therapeutic effects as well as side effects of artemether on diabetic nonhuman primates in vivo in the current study.

## 2 MATERIAL AND METHODS

**FIGURE 1:**
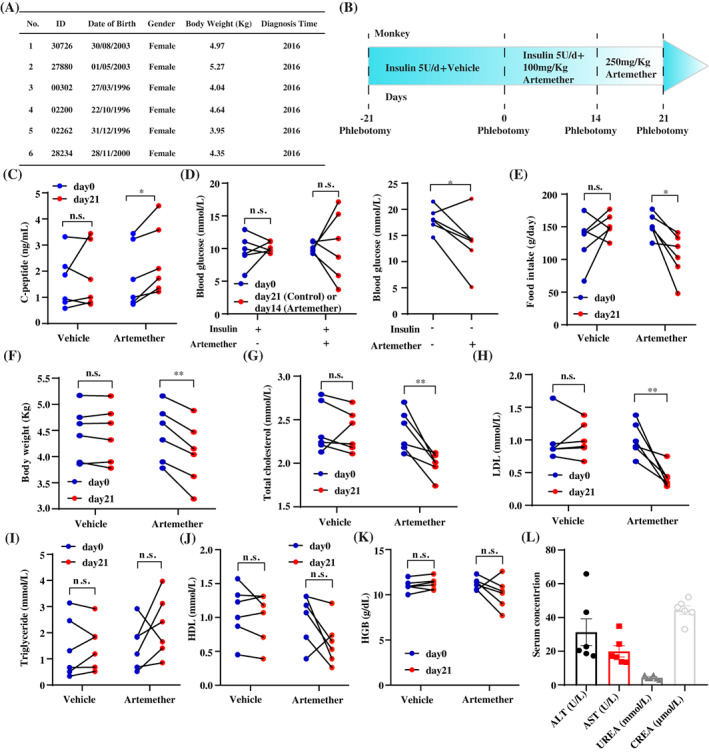
Artemether improves islet function and metabolic homeostasis. (A) Basic information about the animals; (B) Scheme of the experiment; (C) Serum concentration of c‐peptide; (D) Fasting blood glucose with (left) or without (right) insulin treatment; (E) Body weight; (F) Food intake; (G–J) Serum concentration of total cholesterol, low‐density lipoprotein cholesterol (LDL), triglyceride, and high‐density lipoprotein cholesterol (HDL); (K) Serum concentration of hemoglobin (HGB); (L) Serum concentration of alanine transaminase (ALT), aspartate aminotransferase (AST), urea, and creatine (crea) on day 21.

The basic information of the *cynomolgus macaques* is presented in Figure 1A. The experiments were performed on September 2019. This study was approved by the ethical committee of Fudan University (No. 201907002Z). All the monkeys were diagnosed with spontaneously occurred type 2 diabetes. None of the animals were used in any pharmacological experiments before, though they received long‐acting recombinant insulin treatment 5 U/day regardless of the body weight (Ganli Pharmaceutical Industry Co., Taizhou, China) to prevent hyperglycemia. Licensed veterinarians took care of the animals throughout the whole experimental process. No abnormalities, except the behavior related to food intake, were identified in the study.

To minimize the number of animals used in the experiment, before‐after study in the same animals was performed via oral gavage of vehicle or artemether (Figure 1B). Before the experiment started, the animals were trained for 3 weeks so they were willing to receive oral gavage and phlebotomy. The baseline concentration of fasting blood glucose, without insulin treatment, was measured before the experiment started. The animals were treated by the vehicle, which is 1% methylcellulose, for 21 days. Then they were treated by 100 mg/kg artemether for 14 days and 250 mg/kg artemether for another 7 days. After day 14, insulin treatments were stopped to evaluate the therapeutic effects of artemether. Standard diet (Guolong Tech, Guangzhou, China) was provided for the animals ad libitum. According to the ethical regulation, steamed cakes were provided as snacks for the animals that presented low appetite. The amount of food intake presented in this study is the total weight of the standard diet and snacks. The blood was collected at day −21, day 0, day 14, and day 21. The clinical parameters were measured by Roche E411 and C311 analyzer. Paired Student *t* test was used for this study.

## 3 RESULTS

We observed the elevation of serum c‐peptide concentration with 21‐day artemether treatment, which indicated the improvement of islet function (Figure 1C). Artemether did not induce significant changes for the level of fasting blood glucose in the combination of insulin treatment (day 0‐‐day 14). In contrast, the fasting blood glucose level at day 21 without insulin treatment was significantly lower than the baseline level (Figure 1D), which highlighted the therapeutic effects of artemether for hyperglycemia.

We further explored the effects of artemether on the other metabolism‐related phenotypes. Interestingly, artemether treatment led to decreases of food intake and body weight (Figure 1E,F). We also discovered the downregulation of serum total cholesterol and low‐density lipoprotein cholesterol but not total triglycerides and high‐density lipoprotein with artemether treatment (Figure 1G–J). These metabolism‐related phenotypic changes were consistent with what have been reported with rodent diabetic models in the literature.

One major concern of applying artemisinins in patients with diabetes, despite being approved drugs, is whether their long‐term administration will lead to liver or kidney damage. Thus, we measured the serum concentration of hemoglobin as well as liver damage‐related markers alanine transaminase/aspartate aminotransferase and kidney damage‐related markers urea/creatine (Figure 1K,L). The level of all these factors was either not significantly changed by the treatment or within the physiological ranges, which demonstrated that the usage of artemether on primates for 21 days would not induce severe damage on reticulocytes, liver, or kidney.

## 4 DISCUSSION

In the current study, we discovered that artemether treatment relieved the insulin insufficiency, hyperglycemia, and metabolism disorder in diabetic nonhuman primates. The phenotypic changes we observed in this study agreed with most of the changes in rodent diabetic models. Because of the ethical concerns, we did not try to obtain samples of the pancreas. Therefore, we are unable to explore the detailed mechanism for the artemether induced improvement of islet function. It is possible that mechanisms improving β cell function, other than β cell regeneration, contributed to the therapeutic effects of artemether.

The more important information delivered by our study is that artemisinins may decrease food intake in primates. Particularly, three animals were diagnosed as “K2‐refuse to eat” several times when they were treated with 250 mg/kg but not 100 mg/kg artemether. Obviously, “K2‐refuse to eat” is a dangerous side effect. Because the increases of endogenous insulin production by artemether treatment should upregulate food intake and body weight, the “K2‐refuse to eat” is likely a phenotype independent of the improvement of islet function. Therefore, physicians who would like to perform clinical trials for long‐term administration of artemisinins should carefully choose the doses of drugs and monitor the food intake of the participants. In addition, the decrease of food intake may also contribute to the therapeutic effects of artemether on the improvement of islet function and metabolic homeostasis. A study on nonhuman primates with pair‐feeding needs to be performed to further test the causal relationship between artemether treatment and the changes of islet function.

Owing to the technical limitations of the animal facility, we are unable to collect the data about drinking water, urine, and the motility of gastrointestinal tract. As we are not allowed the obtain the pancreas samples, it is impossible to evaluate the changes of islets. This study is also limited by the low number of animals and gender bias. In addition, the animals involved in this study are relatively old, but the patients who are going to receive therapies to improve islet function are generally young. Clinicians carrying on clinical trials of artemether on diabetes should take these factors into consideration.

In summary, our study on nonhuman primates identified the possibilities of artemisinins to be used as drugs for diabetes. The encouraging results derived from this study pave the way to validate the effects of artemisinins in clinical trials in future. We have registered a clinical trial to test the therapeutic effects of dihydroartemisinin on type 2 diabetes (ChiCTR‐IPR‐17010789).

## AUTHOR CONTRIBUTIONS

Conceptualization, investigation, analysis, writing, and data visualization: Wei Fu, Jiang Hongwei, and Jin Li. Funding acquisition: Jiang Hongwei and Jin Li. Supervision: Jiang Hongwei and Jin Li.

## DISCLOSURE

The authors claim no conflict of interests.

## Data Availability

The raw data can be provided by Dr. Jin Li upon reasonable requests.
